# Bias in natriuretic peptide-guided heart failure trials: time to improve guideline adherence using alternative approaches

**DOI:** 10.1007/s10741-020-10004-6

**Published:** 2020-08-12

**Authors:** Susan Stienen, Ankeet Bhatt, João Pedro Ferreira, Muthiah Vaduganathan, James Januzzi, Kirkwood Adams, Jean-Claude Tardif, Patrick Rossignol, Faiez Zannad

**Affiliations:** 1 INSERM, Centre d’Investigations Cliniques Plurithématique 1433, INSERM U1116, Université de Lorraine, CHRU de Nancy, F-CRIN INI-CRCT (Cardiovascular and Renal Clinical Trialists), Université de Lorraine, Nancy, France; 2 Department of cardiology, Amsterdam University Medical Centers, Amsterdam, The Netherlands; 3 Brigham and Women’s Hospital, Boston, MA USA; 4 Department of Physiology and Cardiothoracic Surgery, Cardiovascular Research and Development Unit, Faculty of Medicine, University of Porto, Porto, Portugal; 5 Massachusetts General Hospital, Boston, MA USA; 6 University of North Carolina, Chapel Hill, NC USA; 7 Montreal Heart Institute, Université de Montréal, Montréal, Canada

**Keywords:** HFrEF, Biomarker-guided trials, Biomarker, Natriuretic peptides, Guideline-directed, Medical therapy, Adherence, Bias

## Abstract

Treatment of patients with heart failure with reduced ejection fraction (HFrEF) with currently available therapies reduces morbidity and mortality. However, implementation of these therapies is a problem with only few patients achieving guideline-recommended maximal doses of therapy. In an effort to improve guideline adherence and uptitration, several trials have investigated a biomarker-guided strategy (using natriuretic peptide targets in specific), but although conceptually promising, these trials failed to show a consistent beneficial effect on outcomes. In this review, we discuss different methodological issues that may explain the failure of these trials and offer potential solutions. Moreover, alternative approaches to increase heart failure guideline adherence are evaluated.

## Introduction

Heart failure (HF) is a highly prevalent condition, with an incidence that continues to rise, driven largely by an improved survival of patients with atherosclerotic vascular disease, increasing rates of diabetes and obesity, and aging of the population [1]. Approximately 50% of patients with a documented history of HF have reduced ejection fraction (HFrEF), which is associated with a high risk of mortality and morbidity [2]. Large outcome trials have demonstrated that pharmacologic treatments such as inhibitors of the renin-angiotensin-aldosterone system (angiotensin-converting enzyme inhibitors (ACEi) and angiotensin receptor blockers (ARB) with or without neprilysin inhibitors), beta-adrenergic blockers (BBs) and mineralocorticoid receptor antagonists (MRAs) decrease mortality and hospitalizations rates in HFrEF [3]. However, in clinical practice, use of all of these three drug classes is uncommon and even fewer patients achieve guideline-recommended maximal doses of HF therapy [4, 5]. Previous studies have shown that barriers to HF medication titration include patient-related factors (such as age, sex, body-mass index, renal function, comorbidities, and polypharmacy) but also physician-related (inexperience and knowledge gaps, fear of side effects, therapeutic inertia) and system-related factors (limited time and support structures to facilitate regular monitoring and transitions between hospital and general practitioner) [6–9]. In order to improve treatment adherence and uptitration, it has been hypothesized that a biomarker-targeted strategy could have a role in guiding intensification of guideline-directed medical therapy (GDMT).

## The rationale for biomarker-guided trials

Similar to other therapeutic areas such as hyperlipidemia, type 2 diabetes mellitus, and obesity, measurement of circulating biomarkers has been proposed in an attempt to understand (1) residual risk and (2) response to therapy. For example, LDL cholesterol has been incorporated into guideline recommendations as a target to guide therapeutic efficacy and the need for therapeutic modification. In HF, many markers have been proposed, of which the natriuretic peptides (B-type natriuretic peptide (BNP) and N-terminal pro-BNP (NT-proBNP)) are the most extensively studied. Natriuretic peptides are indicators of ventricular wall stretch [10] and, to date, important parameters for diagnostic and prognostic purposes in HF [3]. Natriuretic peptides reflect congestion but also mirror other mechanisms of HF as can be appreciated by their reduction after initiation of several HF therapies [11]. Moreover, reductions in natriuretic peptides during both the acute and chronic phases of HF have been associated with an improved prognosis [12, 13]. Hence, it was hypothesized that natriuretic peptides allow for an objective assessment of HF severity and may be used to “guide” the initiation and appropriate titration of HF therapies, potentially translating into improved clinical outcomes. As a result, several randomized controlled trials have been conducted in the past two decades assessing a natriuretic peptide-guided strategy and compared with standard of care [14–16]. Unfortunately, such strategies, while conceptually promising, have generally not shown a consistent improvement in outcomes.

## Why have natriuretic peptide-guided HF trials failed thus far?

An overview of current natriuretic peptide-guided HF trials is listed in Table 1. In 5 out of 14 trials, natriuretic peptide-guided therapy reduced the rates of the primary endpoint compared to standard of care. These trials were all characterized by a significant titration of HF therapy in the natriuretic peptide-guided compared to the standard of care arm. Results from these trials suggest that HF therapies, when titrated to guideline recommended doses, lead to significantly lower natriuretic peptide levels and are associated with improved clinical outcomes. However, these positive trials have generally been limited by small sample sizes (ranging between 30 and 120 patients per treatment arm), which may lead to bias in the estimation of the treatment effect. For example, in the PROTECT trial [27], a sample size calculation based on an estimated relative risk reduction in total burden of cardiac events of 40% with NT-proBNP-guided treatment on top of standard of care was performed, while large trials comparing high- versus low-dose renin-angiotensin-aldosterone system (RAAS) inhibitors only demonstrated an ~ 10% reduction in endpoints using a time-to-first-event analysis [28, 29]. The largest trial thus far, the Guiding Evidence Based Therapy Using Biomarker Intensified Treatment in Heart Failure (GUIDE-IT), failed to demonstrate significant differences between patients randomized to NT-proBNP-guided therapy compared to standard of care in medication prescription rates and doses, change in NT-proBNP and the primary endpoint [15]. For this trial, a sample size calculation was performed based on a relative risk reduction of 20% in the primary endpoint (i.e., a composite of time-to-first HF hospitalization or cardiovascular mortality at 12 months).

**Table 1:** Overview of natriuretic peptide-guided heart failure trials

Studies	Inclusion period	Population and centers	NP target	Clinical target	Primary endpoint	Treatment algorithm	Follow-up	Sample size and statistical power	Event rates	NP target attained? (or sign. reduction?)	Medication different at end of FU?	Positive trial?
Anguita [17]	2006–2008	Chronic HF Spanish university hospital (*N* = 1)	BNP < 100 pg/mL	Framingham HF score < 2	Composite ACM and CV hosp	BNP: therapy intensified to achieve target; control: therapy intensified to achieve score	18 months	*N* 30 (conv)/30 (BNP) Power 80%	Not described	✔	✖	✖
Northstar [18]	2005–2009	Chronic HF Danish public HF clinics (*N* = 18)	No target	Standard care	Composite ACM and CV hosp	BNP: evaluate intensification of therapy when > 30% NT-proBNP increase; control: standard care	2.5 years	*N* 208 (conv)/199 (BNP) Power 80%	41% (conv) vs. 45% (BNP)	✖	✖	✖
Starbrite [19]	2003–2005	Chronic HF US university hospitals (*N* = 3)	BNP at discharge	Congestion score	Composite 90-day survival and hospital-free survival	BNP: therapy intensified when BNP > 2× BNP at discharge; control: intensify treatment to achieve target congestion score	3 months	*N* 65 (conv)/65 (BNP) Power 84%	80 ± 21 days (conv) vs. 85 ± 12 days (BNP)	✖	✔ (BB and ACE/ARB + BB)	✖
Upstep [20]	2006–2009	Chronic HF Swedish hospitals, experienced in HF (*N* = 29)	< 75 years: BNP < 150 pg/mL; ≥ 75 years: < 300 pg/mL	Standard care	Composite ACM and hosp. and worsening HF	BNP: therapy intensified to achieve OMT; control: standard care	> 48 months	*N* 132 (conv)/147 (BNP) Power 80%	Not described	?	✖	✖
Christchurch [21]	1998–1999	Chronic HF HF outpatient clinic in New-Zealand (*N* = 1)	NT-proBNP < 1700 ng/mL	Framingham HF score < 2	Total CV events: ACM, hospital admission, new HF-related outpatient episode	BNP: therapy intensified to achieve target; control: therapy intensified to achieve congestion score	6 months	*N* 36 (conv)/33 (BNP) Power: 80%	54 (conv) vs. 19 events (BNP)	✖/✔ (?)	✔ (ACE + MRA)	✔
TIME-CHF [22]	2003–2006	Chronic HF Swiss and German hospitals (*N* = 15)	< 75 years: NT-proBNP < 400 pg/mL; ≥ 75 years: < 800 pg/mL	NYHA ≤ 2	Hospital-free survival	BNP: therapy intensified to achieve target; control: therapy intensified to achieve NYHA ≤ 2	18 months	*N* 248 (conv)/251 (BNP) Power: 80%	40% (conv) vs. 41% (BNP)	✖	✔ (ACE + BB+ MRA)	✖, but sign. Interaction with age
Berger [23]	2003–2004	Chronic HF Viennese university hospitals (*N* = 8)	NT-proBNP < 2200 pg/mL	Standard care	Composite ACM and HF rehosp.	BNP: therapy intensified to OMT; control: standard care	12 months	*N* 90 (conv)/92 (BNP) Power: ?	65% (conv) vs. 37% (BNP)	✔	✔ (ACE + BB, and OMT)	✔
PRIMA [14]	2004–2007	Chronic HF Dutch university and large general hospitals (*N* = 12)	Lowest from discharge BNP or after 2 weeks of FU	Standard care	Survival and hospital-free survival	BNP: therapy intensified to achieve target; control: standard care	1 year	*N* 171 (conv)/174 (BNP) Power 80%	ACM: 33% (Conv) vs. 27% (BNP) Hosp free survival: 664 days (conv) vs. 658 days (BNP)	✖	✔ (ACE + BB)	✖
Signal-HF [24]	2006–2009	Chronic HF Swedish primary care centers (*N* = 45)	> 50% NT-proBNP reduction	Standard care	Composite of ACM, hospital-free survival and symptom score	BNP: therapy intensified to achieve target; control: standard care (both groups received educational program for guidelines)	9 months	*N* 127 (conv)/125 (BNP) Power 80%	Score: 15791 (conv) vs. 14,591 (BNP)	✖	✖	✖
Battlescarred [25]	2001–2006	Chronic HF University hospital from New-Zealand (N = 1)	NT-proBNP < 1300 pg/mL	Framingham HF score < 2	ACM	BNP: therapy intensified to achieve target + congestion score < 2; control: referral to primary physician care	1–3 year	*N* 121 (conv)/121 (BNP) Power: ?	18,9% (conv) vs. 9.1% (BNP)	?	✔ (BB)	✔
Stars-BNP [26]	< 2007	Chronic HF French university hospitals (*N* = 17)	BNP < 100 pg/mL	Standard care	Composite of HF mortality or hosp.	BNP: therapy intensified to achieve target; control: standard care	15 months	*N* 110 (conv)/110 (BNP) Power: ?	52% (conv) vs. 38% (BNP)	?	✔ (ACEi + BB+ MRA)	✔
Protect [27]	2006–2010	Chronic HF US university hospital (N = 1)	NT-proBNP ≤ 1000 pg/mL	Standard care	Composite of worsening HF, HF hosp. and CV events	BNP: therapy intensified to achieve target; control: standard care	10 months	*N* 76 (conv)/75 (BNP) Power: 80%	100 events (conv) vs. 58 events (BNP)	✔	✔ (MRA)	✔
GUIDE-IT [15]	2013–2016	Chronic HF US clinical sites (N = 45)	NT-proBNP < 1000 pg/mL	Standard care	Composite of CV death and HF hosp.	BNP: therapy intensified to achieve target; control: standard care	15 months	*N* 448 (conv)/446 (BNP) Power 90%	37% (conv) vs. 37% (BNP)	✖	✖	✖
PRIMA II [16]	2011–2016	Acute HF Dutch tertiary and secondary HF hospitals (*N* = 9)	>30% NT-proBNP reduction from admission to discharge	Standard care	Composite of ACM and HF hosp.	BNP: therapy intensified to achieve target; control: standard care	6 months	*N* 203 (conv)/202 (BNP) Power 80%	36% (conv) vs. 36% (BNP)	✔	✖	✖

*ACM* all-cause mortality, *HF* heart failure, *NP* natriuretic peptide, *BNP* brain natriuretic peptide, *CV* cardiovascular

Another potential hypothesis for the overall neutral results of the natriuretic peptide-guided trials has been that the majority of the study sites included in these trials had substantial expertise in HF care, possibly reducing between-group differences in HF therapy optimization. Also, it may be that patients were already very well treated at baseline in these specialized HF centers, making a significant change in HF treatment with a natriuretic peptide-guided strategy difficult and the treatment effect smaller. In order to substantiate this, we aimed to compare HF medication use in natriuretic peptide-guided trials versus usual care settings.

## Comparison of HF medication in natriuretic peptide-guided trials vs. usual care

We reviewed the prescription rates and dosages of HF medication (ACEi or ARBs, BBs and MRAs) in 14 natriuretic peptide-guided trials and compared them to recent HF registries (CHECK-HF [4], CHAMP-HF [5], Swedish HF registry [9], QUALIFY [30], and ESC HF long term [31]). In addition, medication differences were studied between patients enrolled in large biomarker-guided randomized clinical trials based on their randomization assignment (natriuretic peptide-guided arm versus standard of care). Medication doses and treatment were compared at baseline and at the end of follow-up for each trial. Given the little evidence for disease modifying therapies in patients with HF and preserved ejection fraction, only patients with HFrEF were considered.

**Fig. 1:**
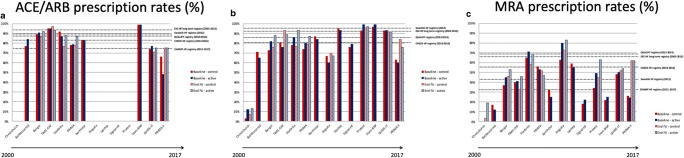
Prescription rates of ACEi/ARBs (**a**), beta-blockers (**b**), and MRAs (**c**) in the different natriuretic-peptide guided-heart failure trials at baseline and end of follow-up according to treatment arm. In PRIMA II, both new onset as chronic decompensated patients were included. Moreover, for these analyses, only patients with a left ventricular ejection fraction below 45% were selected

**Fig. 2:**
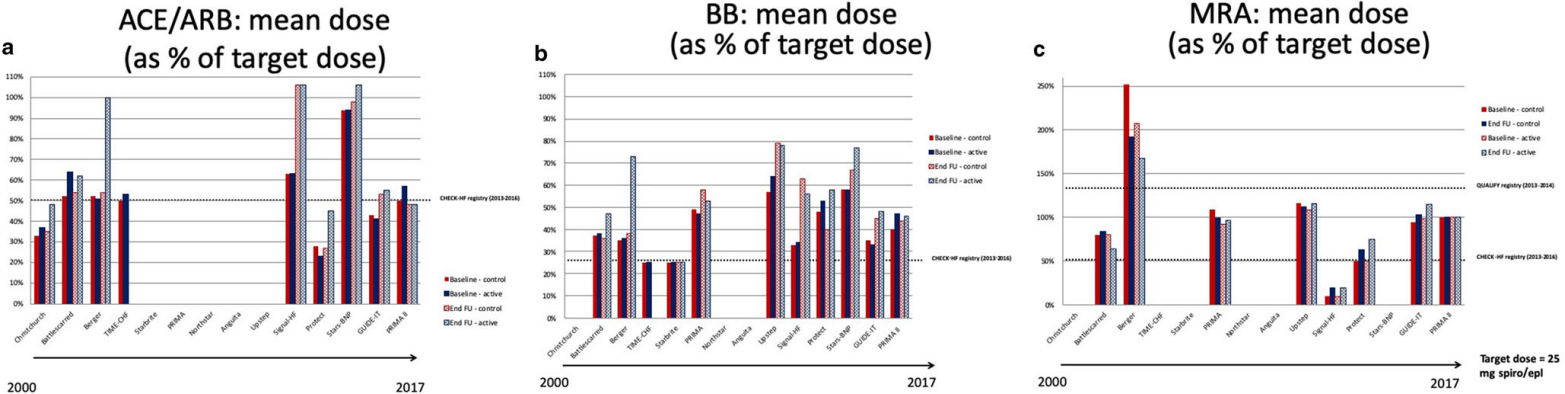
Mean doses (as % of target dose) of ACEi/ARBs (**a**), beta-blockers (**b**), and MRAs (**c**) in the different natriuretic-peptide guided-heart failure trials at baseline and end of follow-up according to treatment arm. In PRIMA II, both new onset as chronic decompensated patients were included. Moreover, for these analyses, only patients with a left ventricular ejection fraction below 45% were selected

Prescription rates of ACEi/ARBs, BBs, and MRAs for all trials and registries are shown in Fig. 1a–c. Mean doses (as percentage of the maximum target dose as recommended by the ESC guidelines) [3] are shown in Fig. 2a–c. Prescription rates for ACEi/ARBs, BBs, and MRAs were low but similar between the natriuretic peptide-guided trials and recent HF registries. These data highlight the continued opportunity for improvement in GDMT implementation in patients with HF, and refute the notion that enrolled patients are already well treated for HF.

However, in natriuretic peptide-guided trials, HF medication was titrated to doses higher than those observed in a recent registry [4] irrespective of randomization to natriuretic peptide-guided therapy or standard of care (Fig. 2). These findings suggest that investigators in natriuretic peptide-guided trials titrated HF medication to a similar extent in the natriuretic peptide-guided and the standard of care arms. But how do we reconcile these findings? A comparison of baseline characteristics of patients included in the trials and registries showed that patients generally have a similar age, distribution of sex, ischemic heart disease, systolic blood pressure and renal function (Table 2). Natriuretic peptide levels were slightly lower in the QUALIFY-HF [30] and ESC long-term HF registry [31] compared to the natriuretic peptide-guided trials, although patients within the Swedish HF registry [9] had similar natriuretic peptide levels as in the trials. This suggests that, in theory, differences in HF medication titration (and dosages) should not be present between the natriuretic peptide-guided trials and HF registries.

**Table 2:** Baseline characteristics of natriuretic peptide-guided trials and HF registries

	Natriuretic peptide-guided trials														HF registries				
	Anguita	Northstar	Starbrite	Upstep	Christ-church	TIME-CHF	Bergen	PRIMA	Signal-HF	Battle-scarred	Stars-BNP	PROTECT	GUIDE-IT	PRIMA-II	CHECK-HF	Swedish HF	QUALIFY	CHAMP-HF	ESC long term
Mean age (years)	70	73	61	71	70	76	72	72	78	76	66	63	63	77	71	75	63	68	66
Female sex (%)	32%	24%	30%	27%	27%	35%	30%	42%	30%	38%	44%	12%	31%	50%	34%	27%	36%	29%	29%
Ischemic etiology of HF (%)	31%	57%	44%		74%	57%	64%	60%		70%	52%	56%	50%	44%	54%	67%	57%	39%	43%
Mean SBP (mmHg)		125	107			119	123	118	134	124		109	114	132	125	122	127	120	120
Mean eGFR		62		61										49	57	61		60	67
Mean creatinine (mg/dL)			1.4	1.2		1.3		1.6		1.4	1.1	1.5	1.3	1.3			1.2		1.1
Median NT-proBNP (ng/mL)		1900			1872	4300	2300	2945	~ 2500	~ 2000		2118	2632	6200		3100	1280		1258
Median BNP (ng/mL)	134		445	850							350								

A potential explanation for the similar HF medication uptitration in both arms of the natriuretic peptide-guided trials may be internal contamination. In this setting, participating in a trial may result in a greater propensity of providers to actively uptitrate therapy. In addition, trial populations may capture a more engaged group of patients, and the infrastructure that exists at tertiary centers may allow for more structured follow-up to facilitate easy uptitration. In contrast, major registries enroll a usual care population with representation from of primary and secondary care centers, which may not have active programs for medication titration but are overall reflective of the HF population at large. The majority of centers participating in the biomarker-guided trials were indeed tertiary centers with specialized heart failure care programs (Table 1). Beyond this, intensity of follow-up related to greater than usual management might compound this hypothesis: in the GUIDE-IT HF trial, patients in the Usual Care arm of the trial were seen on average 10 times in a 15-month period, far more than is typical in usual care settings. In total, those in the usual care arm in GUIDE-IT HF had comparable reduction in NT-proBNP compared to the biomarker-guided arm [32].

**Fig. 3:**
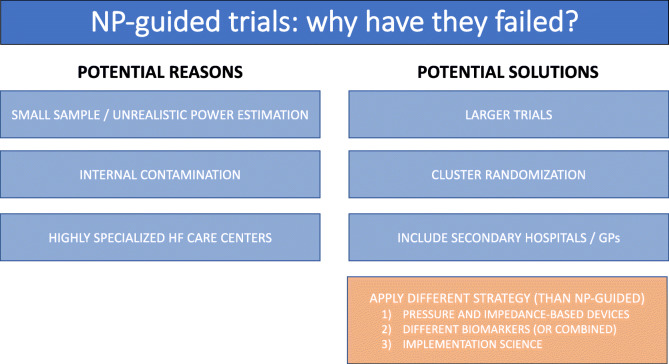
Failure of natriuretic peptide-guided trials: potential reasons and solutions to overcome them

A summary of the potential reasons for failure of natriuretic peptide-guided trials is provided in Fig. 3.

## Alternative approaches to increase HF guideline adherence

There are large opportunities to improve care of heart failure patients by optimizing GDMT. The challenge of providing high-quality, consistent, uptitration will require a unified approach that spans both the inpatient and outpatient settings. This will require the development of new trial strategies focused on implementation and engagement across the healthcare spectrum. Moreover, the efficacy of other biomarker (than natriuretic peptides) and non-biomarker approaches to increase uptitration of HF medication may be tested. We offer some suggestions for the future design of guided GDMT trials in HF (Fig. 3).

### Cluster randomized natriuretic peptide-guided trials

In general, blinding of the study procedure is performed in randomized controlled trials in an effort to minimize contamination. However, in cases where blinding is not possible or not preferred (such as with natriuretic peptide-guided HF treatment), cluster (or group) randomization may be considered. Here, a whole cluster (e.g., a hospital, geographical location, or a single physician) is assigned to a treatment or a control condition. In addition, a cluster design can also have practical advances over individual randomization in the form of lower costs and/or less challenging logistics. A disadvantage is however that a cluster randomized trial requires a larger sample size than a conventional randomized controlled trial as participants cannot be assumed to be independent because of the similarities within the same cluster, resulting in between-cluster variation and the need for adjustment [33].

### Other biomarker/hemodynamic-guided trials

Natriuretic peptides are among the most strongly predictive markers for the diagnosis and prognosis of HF [3]. Suboptimal HF guideline adherence is probably not only due to inertia since this could be overcome by prompt signs of poor health (such as reflected by natriuretic peptides). Other explanations may be an inadequate assessment of residual decongestion and concerns about renal and potassium safety.

Pressure and impedance-based devices and/or biological biomarkers have the potential to identify patients with subclinical residual congestion who might potentially benefit from HF therapy optimization [34]. For example, the CardioMEMS Heart Sensor Allows Monitoring of Pressure to Improve Outcomes in New York Heart Association Class III Heart Failure Patients (CHAMPION) trial demonstrated that pulmonary artery pressure-guided management of chronic HF patients using a wireless implantable hemodynamic monitoring system (CardioMEMS HF System, Abbott, Atlanta, GA) versus standard care significantly reduced HF hospitalizations [35]. While this trial focused on the appropriate management of subclinical congestion and not HF guideline adherence, we could envision a correlate trial with the use of a hemodynamic monitoring device in order to facilitate guideline medication uptitration. This may be a promising further area of study, particularly given angiotensin receptor neprilysin inhibitor therapy has overlapping natriuretic properties, and has been shown to reduce the need of high-dose diuretics [36].

Other promising, non-invasive, techniques are the measurement of lung impedance and lung ultrasonography to detect changes in lung fluid. Heart failure therapy guided by lung impedance measurements has been shown to significantly reduce HF hospitalization rates but again has currently been focused on monitoring subclinical congestion in order to augment diuretic therapy [37]. In a proof-of-concept study, lung ultrasound-guided diuretic treatment of pulmonary congestion also reduced the number of decompensations and improved walking capacity in patients with chronic HF [38].

A surrogate marker of congestion that has been proposed is estimated plasma volume (ePVS), calculated using hemoglobin and hematocrit [39]. Changes in ePVS were associated with cardiovascular endpoints in HF patients [39, 40]. Whether ePVS-guided treatment can improve outcome of HF patients has yet to be determined. Future areas of study may be to apply similar approaches to intensification of GDMT, as uptitration of therapy has been noted to reduce wall stress, improve left ventricular function, and perhaps reduced clinical congestion.

Overall, many of the biomarker and hemodynamic parameters have, to date, been used to detect early congestion and facilitate more aggressive diuresis. Evaluation of these markers and creating a guided strategy for medication uptitration may be useful. In addition, a more routine evaluation of, for example, iron status and QRS duration may lead to (earlier) identification of candidates for iron and cardiac resynchronization therapy respectively. Furthermore, the use of large data sets to define a “poly-biomarker” risk score may provide more utility then a single biomarker or the hemodynamic parameter alone. Such an approach would require the integration of biomarker data in conjunction with hemodynamic parameters in order to both identify patients at risk for decongestion, but also potentially identify opportunities for active uptitration in patients with continued residual heart failure risk. In an in silico analysis from BIOSTAT-CHF, a biomarker-based scenario (in which HF patients would have been uptitrated based on biomarker values) was favorable over a scenario in which all patients would have been successfully uptitrated to > 50% of recommended doses of ACEi/ARB, BB, and MRA [41]. Further study is needed to understand whether a poly-biomarker risk score guided approach may provide a framework for medication uptitration.

Numerous other markers have been identified as possible titration targets, including high-sensitivity troponin and soluble ST2 (STADE-HF (NCT02963272)). However, the impact of guided therapy using these biomarkers on the titration of HF therapy is probably limited since similar methodological (and practical) issues may be expected as were seen for natriuretic peptide-guided therapy.

### Implementation approaches

A variety of non-biomarker approaches to increase HF guideline adherence have been assessed across multiple different settings. A recent observational study [42] using linked primary and secondary care data from general practices in the UK demonstrated that the median time from recorded HF symptoms to an actual HF diagnosis was 972 days (IQR 337–1468) and the time-to-treatment with HF-relevant medication 803 days (IQR 230–1364). It can be subsequently hypothesized that a program targeting an earlier recognition of HF could increase the uptake of guideline adherence, a concept that is currently under investigation [43]. In addition, educational efforts to improve HF therapy are being studied such as a “HF guideline training” for providers [44] or a combined approach at both the physician and patient level [45]. Inclusion of a multidisciplinary carrier team may be critical in facilitating uptitration, including nurse or pharmacist-led titration of HF medications [46, 47]. The concept of self-monitoring and medication self-titration appeared feasible in high-risk hypertensive patients [48] and could potentially be employed in the heart failure setting perhaps utilizing mobile health technologies. Also, the development of GDMT clinics focusing on medication uptitration by highly specialized, well-experienced HF care providers has been suggested [49].

Another area of clear opportunity for therapy optimization is during or immediately following acute hospitalization for HF. It has been previously shown that in-hospital initiation of beta-blockers was safe in patients stabilized after HF admission, with no increased length of stay and with increased use 60 days after discharge compared with those patients without in-hospital initiation [50]. Also, recent data from PIONEER-HF suggests that an in-hospital initiation strategy was safe in patients stabilized after acute HF and may result in important improvements in clinical outcomes at 8 weeks [51]. An intensive, protocol-driven titration of HF therapy after discharge is currently investigated in the “Safety, Tolerability and Efficacy of Rapid Optimization, Helped by NT-proBNP and GDF-15, of Heart Failure Therapies” (STRONG-HF; NCT03412201) study. Enhanced communication strategies to facilitate post-discharge care, including HF transition teams, may improve transitional communication and achievement of target doses of HF medication within 6 months of hospital discharge [52].

All of these efforts are worthwhile; however, the simplest approach would maybe be the development of an algorithm for the identification of patients at risk for not being uptitrated. This algorithm would subsequently create an automatic alert suggesting titration of HF medication. Targeting a high-risk population (i.e., patients after hospitalization for HF) could therefore be a cost-effective option. It is however possible that this population may be too sick to experience benefits from an intensified treatment approach. Moreover, the potential gain that can be achieved when medication adherence is improved across all HF patients should not be underestimated [53].

Excellent therapies are available for the treatment of HF with reduced ejection fraction but it is clear that patients do not receive the best treatment possible. Although several potential explanations can be put forward, we should no longer ignore our (partial) role in the undertreatment of our HF patients. Moreover, the recent discovery of, for example, SGLT2 inhibitors as novel therapeutic agents for HF has been groundbreaking but may unfortunately even further complicate routine clinical practice since this is yet another therapy that needs to be incorporated in a patients’ treatment regimen.

There is a great need for a novel approach to improve HF care, possibly in the form of a dedicated multidisciplinary team having continuous access to telemedicine data including point-of-care biological measurements and pulmonary artery pressures. Moreover, the concept of natriuretic peptide-guided therapy cannot be dismissed before a cluster randomized trial is performed.

## Conclusions

A careful review of literature led us to conclude that natriuretic peptide-guided trials were generally overoptimistic in the sample size estimation and subject to internal contamination bias, as can be observed by a similar titration of HF medication in the intervention and control groups and comparable reduction in NT-proBNP in the pivotal GUIDE-IT HF study. Potential solutions to overcome these issues are to perform a more realistic power calculation and a cluster randomization design. However, other promising approaches to improve HF guideline adherence exist such as pulmonary artery pressure and impedance-guided treatment and a variety of programs aiming at a better implementation of HF care. Further study is needed to identify a strategy enabling the use of GDMT in heart failure patients that is easy to implement in different clinical care settings worldwide.
